# Pollen antigens and atmospheric circulation driven seasonal respiratory viral outbreak and its implication to the Covid-19 pandemic

**DOI:** 10.1038/s41598-021-96282-y

**Published:** 2021-08-20

**Authors:** Michael G. Wallace, Yifeng Wang

**Affiliations:** Sandia National Laboratories, P.O. Box 5800, Albuquerque, NM 87185-0779 USA

**Keywords:** Immunology, Plant sciences, Biogeochemistry, Climate sciences, Environmental sciences, Diseases

## Abstract

The patterns of respiratory virus illness are expressed differently between temperate and tropical climates. Tropical outbreaks often peak in wet seasons. Temperate outbreaks typically peak during the winter. The prevailing causal hypotheses focus on sunlight, temperature and humidity variations. Yet no consistent factors have been identified to sufficiently explain seasonal virus emergence and decline at any latitude. Here we demonstrate close connections among global-scale atmospheric circulations, IgE antibody enhancement through seasonal pollen inhalation, and respiratory virus patterns at any populated latitude, with a focus on the US. Pollens emerge each Spring, and the renewed IgE titers in the population are argued to terminate each winter peak of respiratory illness. Globally circulated airborne viruses are postulated to subsequently deposit across the Southern US during lower zonal geostrophic winds each late Summer. This seasonally refreshed viral load is postulated to trigger a new influenza outbreak, once the existing IgE antibodies diminish to a critical value each Fall. Our study offers a new and consistent explanation for the seasonal diminishment of respiratory viral illnesses in temperate climates, the subdued seasonal signature in the tropics, the annually circulated virus phenotypes, and the northerly migration of influenza across the US every year. Our integrated geospatial and IgE hypothesis provides a new perspective for prediction, mitigation and prevention of the outbreak and spread of seasonal respiratory viruses including Covid-19 pandemic.

## Introduction

The cycles of seasonal respiratory virus (SRV) illness remain widely recognized, but poorly understood. A better understanding of their signatures might lead to improved strategies to combat both seasonal illnesses and pandemics such as the current Covid-19 case. The seasonal virus cycles have long been identified to vary between temperate and tropical climates. Tropical outbreaks often peak in wet seasons, and temperate outbreaks typically peak during the winter^1^. The prevailing causal hypotheses have focused on temperature, sunlight, and humidity variations^1–3^. Yet no consistent factors have been identified to sufficiently explain or predict seasonal virus emergence and decline at any latitude.

Seasonal vaccination campaigns highlight that antibodies (triggered by those vaccines) can be effective inoculants against influenza-like illnesses (ILI). Interestingly, much like any SRV vaccine, the antigenic reaction triggered by pollen is also accompanied by the production of antibodies^4^. The well-known IgE antibody emergence over pollen season appears to affect all who are exposed, whether or not they develop allergies^5^. These antigenic reactions from pollen are not widely credited for diminishing ILI, perhaps because the associated IgE antibodies are not found in significant concentrations in human subjects other than during pollen season. Yet cross reactivity of antibodies is a common research area, and some publications have identified this attribution for IgE to counter SRVs^6,7^ as well as potentially for the treatment of tumors^8^. This suggests that although larger than viruses, pollens trigger IgEs and, once the IgE titers in a population rise, this antibody type might also target SRVs.

Accordingly it is of interest to examine the seasonalities of ILIs and of pollens as well as any possible relations to the antibodies that pollens antigenically trigger in humans. This investigation features a geospatial examination of the seasonal atmospheric circulations of both pollens and viruses. As aerosol and near-aerosol sized particles, the circulations of these components can be examined from data and characterized by routine flow and transport principles.

**Figure 1:**
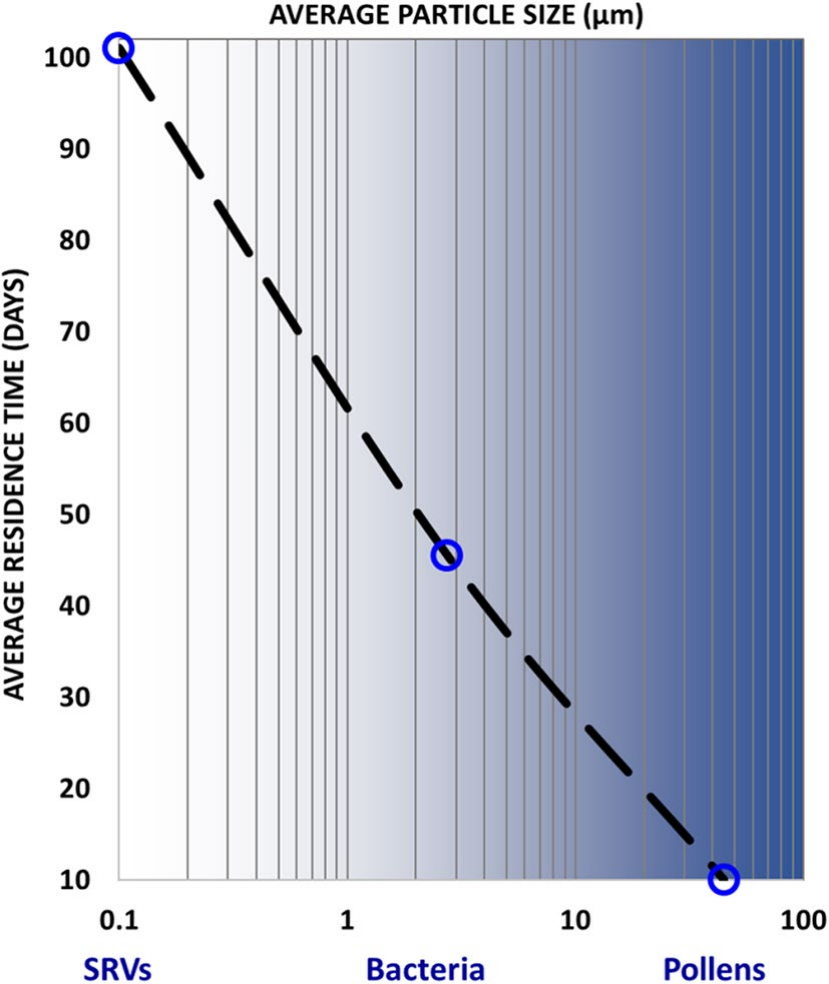
Average residence time of atmospheric particles as a function of particle size. Adapted from Table 1, Anastasio and Martin^17^ and Kreidenweiss et al.^18^.

It has been suggested that viruses as drifting “accumulation mode” particles could be widely distributed through the atmosphere and could circulate in part according to their sizes^9^. We recognize that this conceptualization is consistent with the Navier Stokes formulation of fluid flow and particle transport. The vertical settling velocity of any small particle as described by the Stokes equation (Eq. 1, described subsequently) is also countered by the lift of particles as a function of the wind speed via the Reynolds number^10^. Typical atmospheric residence times of bioaerosols including viruses and pollens are outlined in Table 1 and Fig. 1. Wet removal of aerosol particles through atmospheric precipitation is illustrated by the graded blue shading. The susceptibility of a particle to washout increases with its particle size. Given average dimensions of 0.1 micron, viruses are more prone to lift and less prone to sedimentation than larger particles. As a result, their environmental range extends from the surface to the region above the Planetary Boundary Layer (PBL), approximately 1000 m above the Earth’s surface. Accordingly, viruses exhibit the longest atmospheric residencies among the categories considered. Given that atmospheric velocities typically range from a few meters per second slightly above the planet’s surface to 70 m per second above the PBL, viruses lofted high in the atmosphere along certain tracks are expected to circulate globally around the Earth several times a month. In contrast, pollen dimensions are several orders of magnitude larger, ranging roughly from 10 to 100 microns, and accordingly have much lower atmospheric residence times. These particles would rapidly settle out of the atmosphere simply by gravity and rainfall. As noted, pollens are of significant interest in this investigation due to indications that they possess immunological properties against seasonal respiratory viruses SRVs^7^.

**Table 1:** Estimated aerosol settling velocities and residence times.

Particle	Estimate of average diameter (µm)	Stokes settling velocity m/yr (Eq. 1)	Residence time (days) (Fig. 1)
Small influenza virus	0.1^11^	3	100
Large coronavirus	0.2^12^	17	90
Large measles virus	0.3^13,14^	37	80
Bacteria	2.8^15^	3250	46
Pollen	42^16^	7.3E+05	10

No seasonal ILI studies to date have considered pollen in concert with global circulation of viruses in the atmosphere. Agent-based transmission from host to host remains the primary conceptual model for virus spread, as demonstrated in the widespread use of Susceptible, Exposed, Infectious, and Recovered (SEIR) models^19^. Some of these models have considered the atmospheric survivability of SRVs. For example, recent work^1,3^ proposed that humid air diminishes transmission of an influenza virus in temperate zones. Yet rainy seasons in tropical regimes are also humid seasons, and those can be the times in those latitudes when influenza is most prevalent.

Other studies that factored in the atmospheric transport of bioaerosols sought to explain episodes of sudden and extensive viral outbreaks covering vast regions^9^. Whon et al.^20^ first reported the seasonality of airborne viruses and their genetic diversity across regions of South Korea. Recent investigations by Reche et al.^21^ further demonstrated the exceptional variety and intensity of viral flux from above, through the PBL at two sites within Spain’s Sierra Nevada Mountains. Although the circulation of viruses around the planet is no longer controversial, the application of this knowledge to better understanding SRV outbreak and spread has not been extensively explored. Some work has pointed out intriguing seasonal and geographic patterns of flu onset across tropical locations including Brazil^22–24^. As noted in Tamerius et al.^23^, the rainiest periods across Brazil highly correlate to flu outbreaks. Moreover, the flu season is described to originate at the northern boundary of Brazil in the tropical latitudes by April and migrate towards the south, in the subtropical latitudes over several months every year^24^. Those studies also assume an agent-based transmission paradigm, and accordingly ILI patterns were believed to initiate or intensify from a weather feature but not spread further due to climate. Furthermore, both the World Health Organization (WHO) and the Centers for Disease Control and Prevention (CDC) have on occasion noted a geospatial pattern of south to north migration of flus in temperate climates^25,26^. That pattern is accordingly somewhat symmetric to the reported Brazilian ILI pattern. A confirmation of this migration direction within the US is found in Charu et al.^27^ where the authors noted “seven of eight (ILI) epidemics likely originated in the Southern US”.

A consistent and reproducible model for understanding SRV outbreaks is essential for the prediction, mitigation and prevention of ILIs including the Covid-19 pandemic. Herein we explore correlations between ILIs and key seasonal climatological variables, and we identify a possible causal correlation between the emergence and migration of ILIs with the seasonal emergence and decline of pollens. These analyses are based primarily on data collected from two Western Hemisphere regions, the United States (including Albuquerque, New Mexico) and Fortaleza, Ceará, Brazil.

## Results

**Figure 2:**
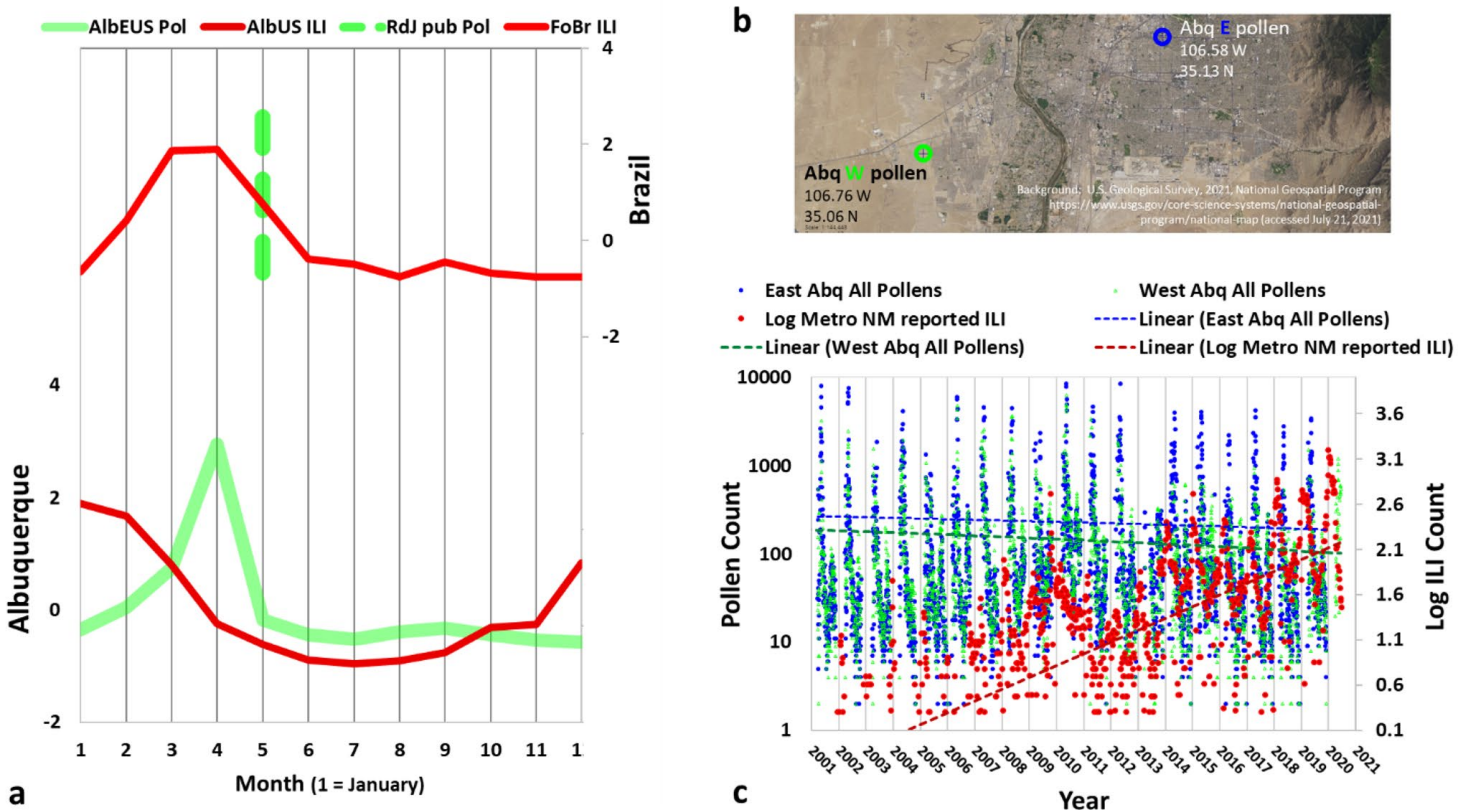
(**a**) Average monthly (climatological) patterns for ILI (red) and pollen (green) for Brazil (top) and Albuquerque, US (bottom). The data are averaged from 2001 to the present. The dotted green line, indicating the occurrence of pollen peak, is based on Greco et al^29^. The solid lines are based on data from Tamerius et al.^23^ for Brazil ILI and NMDOH^30^ for Albuquerque ILI (both red) and from CABQ^31^ for pollen (green). (**b**) Locations of Albuquerque pollen monitor stations. (**c**) Comparison of reported ILIs to records of two pollen monitoring stations for Albuquerque, NM, US. Sources ILI^30^ pollen^31^.

The results presented below were derived primarily from geospatial data compilation and analyses of atmospheric (including geostrophic) resources along with ILIs, and pollen data (see Methods in SI). For consistency we explored temperature and precipitation variables based on both surface and geostrophic monthly data. Although pollen monitoring contributes high resolution historical data at many locations, there is no central public pollen database. Accordingly, a handful of papers and isolated data resources are profiled here to expand upon notions of airborne pollen occurrence across seasonal domains in both temperate and tropical regions. For example, Behling and Negrelle^28^ relates tropical pollens to rainfall, and Greco et al.^29^ notes several years of pollen monitoring results near Rio De Janeiro, Brazil. The resulting pollen spike occurs in May. We rely upon this work of Greco et al.^29^ as a proxy for the Fortaleza location as displayed by the dotted green vertical line in Fig. 2a.

With the exception of cyclonic events, winds are routinely stronger in temperate zones than tropical ones. Figures S1 through S5 provide example geostrophic wind maps, which demonstrate the generally lower velocity wind patterns by the dark blue shading. The tropical regions are accordingly known for “pollen rain”, where pollen falls directly to the ground from the overlying tree canopy. Pollen rains explain why regional pollen collection in tropical zones are challenging^28^. In contrast, temperate Albuquerque’s two pollen stations (mapped in Fig. 2b) provided an extensive record of a wide variety of wind-mediated pollens. Moreover, the influenza surveillance for that city covered a similar time span and resolution. As shown in Fig. 2c, two pollen time series (blue and green dots) and one ILI series (red dots), were accordingly developed based upon resources provided directly by the New Mexico Department of Health^30^ for ILI, and the City of Albuquerque^31^ for pollen. As indicated in Fig. 2b, the two Albuquerque pollen stations were placed to monitor environmentally distinct locations, one for the west mesa region and the other in the residential northeast, near the base of the Sandia Mountains. The west station is more exposed to westerly winds crossing unirrigated lands and has lower pollen levels (green) than the urban eastern pollen station (blue). By virtue of the general arid environment, both series show lower ranges of pollen than many other sites throughout the wetter central and eastern US. Figure S6a charts raw Albuquerque time series for a selection of pollen types. Figure S6b further complements the ILI patterns over time by the addition of ILI series representing NM’s quadrants. The NM ILI information is also shared with the US Centers for Disease Control and Prevention (CDC) in the development of their “FLUView” ILI dashboard^32^. Values of ILIs are commonly represented as both numbers of positive cases and as the unweighted percentage of those numbers in comparison to the total number of health care provider—reported illnesses in each locale.

**Figure 3:**
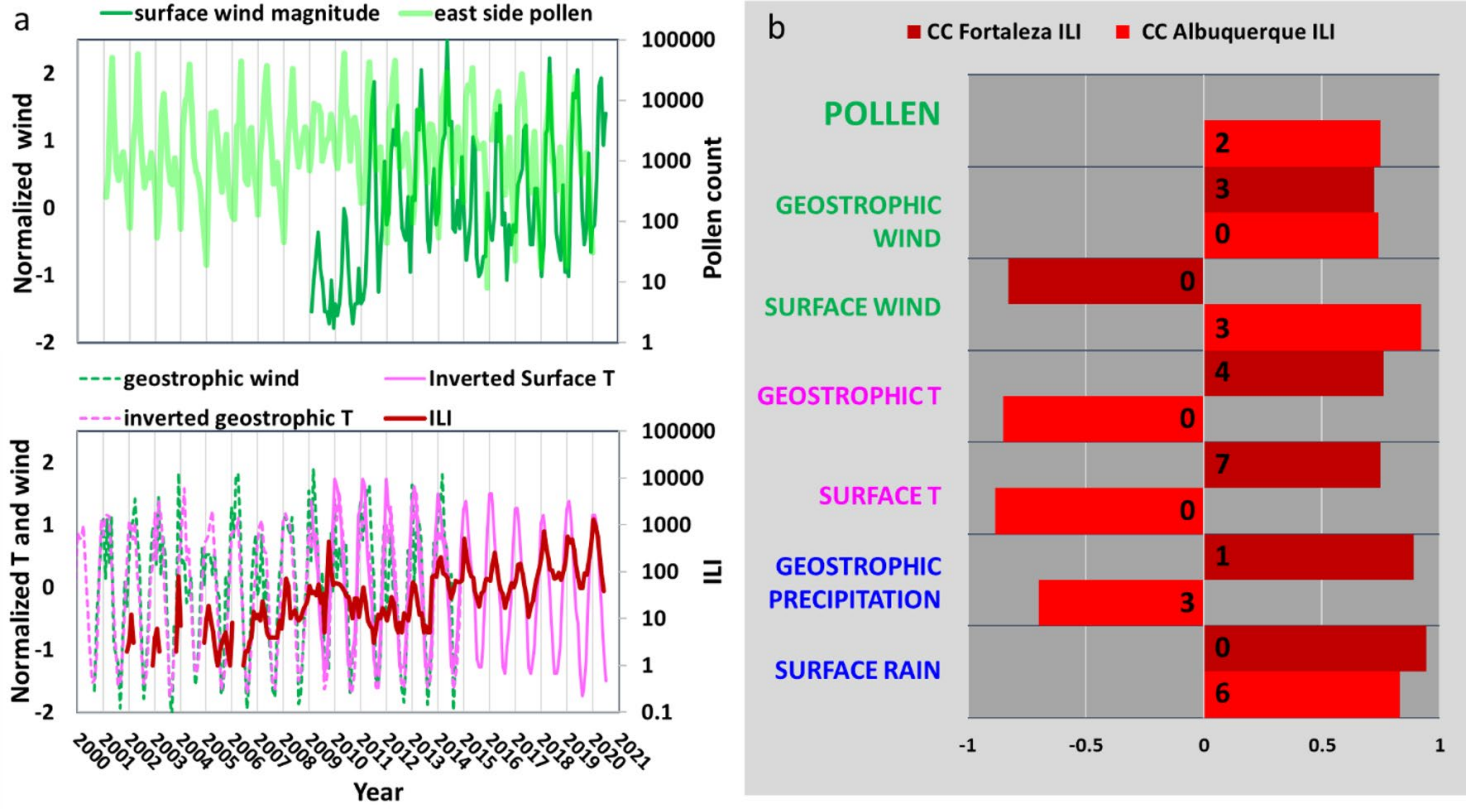
Time series and correlations of featured climatological variables and ILI. (**a**) Top panel: East Albuquerque pollen counts^31^ and normalized surface winds^34^ over the past two decades. Bottom panel: Albuquerque ILI count^30^ and normalized geostrophic wind^35^, and geostrophic^35^ and surface^34^ temperature over the same time period. (**b**) Correlation coefficients (cc) of seasonal variables to Albuquerque ILI (bright red) and Fortaleza ILI (dark red). The Methods section in SI covers the general and geostrophic development of these charts.

**Figure 4:**
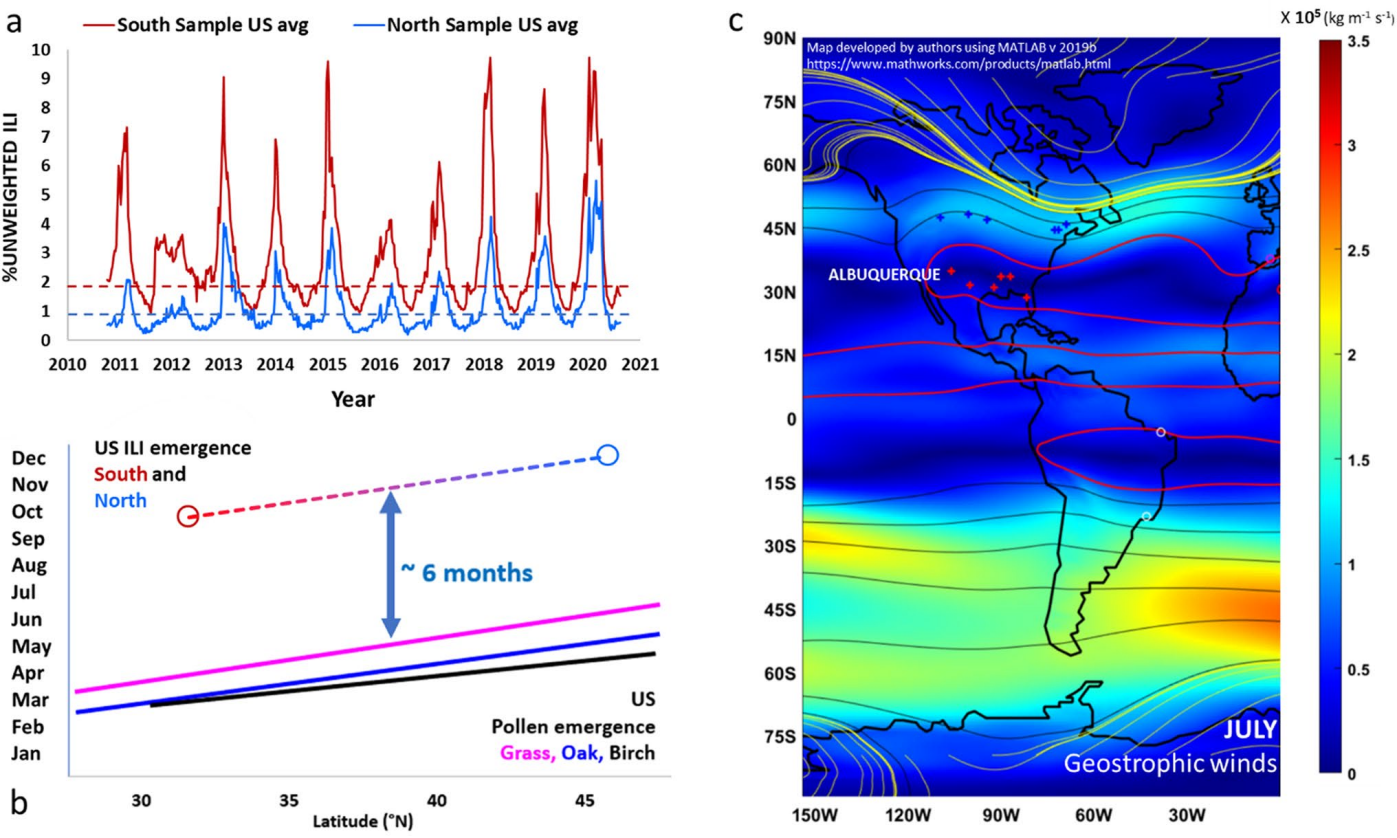
Comparison of ILI, pollen and geostrophic wind migrations. (**a**) Comparison of southern state ILIs averages to the northern state reported unweighted ILIs. Unweighted ILIs are the percentage of ILI diagnoses in comparison to all illness diagnoses from each sentinel site^32^. (**b**) Emergence dates for ILI and pollen across the US. (Adapted from references^32,36^) (**c**). Geostrophic winds^35^ with streamlines in July. Black lines originate from 0E. Red lines originate from 180W Yellow lines originate from 80N and 80S.

The thin dotted lines in Fig. 2c indicate that pollens have trended slightly down over the two decades profiled, while ILI cases have risen significantly. This decadal upward trend in ILI (Figs. 2c, 3a and S6b) can also be observed in other regions within the US (as indicated subsequently in Fig. 4a), thus appearing to be a large-scale phenomenon. In contrast, no consistent trends have been observed for pollens^33^. Given the size and source of pollen particles (Fig. 1), their concentration trends are likely to be controlled by local environmental factors.

The data from Albuquerque, US and Fortaleza, Brazil clearly indicate a close association of pollen emergence and decline with viral outbreak episodes in both climate regimes (Fig. 2). Just as with ILIs, airborne pollen concentrations rise and fall exponentially within any given year, with each ILI peak occurring between two successive pollen peaks. Other seasonal variables, both surface and geostrophic, rise and fall significantly across any year (Fig. S7a,b). For both locations, Evaporation minus Precipitation (EP) for the full atmosphere (“geostrophic rain”) closely follow the surface rainfall (Fig. S7), thus adding confidence to the use of geostrophic information in correlating weather to SRVs. As shown in Fig. S7a and b, many aspects of the Albuquerque and Fortaleza climatological curves are similar when factoring in a two-to-three-month shift in general, as well as the North American Monsoon (NAM) impact on rainfall in Albuquerque. Pollen emergence coincides with the decline of viral spread in both climate regimes (Fig. 2a). Pollen concentrations decline in temperate climates during the late fall and winter because pollens are not produced then. Over heavy rainy seasons in tropical zones, airborne pollens are also diminished due to washout.

As discussed above, while viruses can be transported by upper atmosphere circulation over extended periods, pollens are expected to be controlled by local climatological factors. This is supported by Albuquerque data. Figure 3a and b show the comparisons of surface winds to pollen concentrations (top panel) and of ILI to geostrophic winds (bottom panel). In these figures the raw datasets were interpolated into weekly increments to cover available spans up through the past two decades. Figure 3a confirms an anticipated synchrony between surface winds (solid green curve) and pollen loads (transparent green curve), and a closer synchrony for the final decade. Figure 3b shows an equally strong synchrony between ILI percentages (red curve) and geostrophic winds (dotted green curve) as well as inverted temperatures, both geostrophic and surface (dotted and solid pink curves, respectively).

Figure 3b summarizes the correlation analysis of pollens, winds, temperatures and precipitations to monthly ILIs at both Albuquerque (bright red) and Fortaleza (maroon) using the Pearson’s method. The monthly lags for each correlation are indicated in black. Because all variables are normalized first and there are 12 entries per variable for each test, any R value greater than 0.5 is statistically significant. It is not surprising that SRVs and each category of weather and pollen variable are correlated, but that the strength and direction of the correlation relations can vary between temperate and tropical locations. Tamerius^23^ indicated a high correlation between ILIs and rainfall at Fortaleza, which is confirmed in Fig. 3b by the high correlation coefficients of geostrophic and surface rain to ILIs with no time lag. For Albuquerque, the low lag high correlations (whether positive or negative) were found for winds and temperatures. Precipitation is also found to correlate, but only at certain lags. This is attributed to the NAM which reaches Albuquerque every late summer. For Albuquerque pollen, although the peak month occurs two months after the peak ILI month, the diminishment of ILIs and the emergence (as opposed to the peak) of a high pollen level are highly correlated practically with no time lag, both for Albuquerque and Fortaleza (Fig. 2a). Moreover, the figure demonstrates a 6 month lag between peak pollen and the subsequence re-emergence of ILI for both locations. It is reasonable to expect that any primary factor controling virus seasonality would exhibit a consistent correlation with ILIs for both locations. Accordingly correlations would express either the same signs or the opposite signs with a time lag of ~ 0 or 6 months. From Figs. 2a and 3b, only three factors are identified to be possible candiates: pollen, geostrophic wind, and surface temperature. As shown below, the surface temperature can further be eliminated.

A seasonal pattern of south to north migration of influenza in temperate climates was described earlier to have been observed but never explained. To analyze further, we examined ILI time series for two bands of southern and northern states in the US and compared the emergence times of ILI to the emergence times of pollen in equivalent latitudes.

States east of the continental divide which are best aligned to the northern and southern boundaries of the US were identified, and ILI information was developed accordingly from the CDC’s FLUView resource^32^. States featured from the south and identified by the red curve in Fig. 4a and the red crosses in the geostrophic wind map of Fig. 4c were New Mexico, Texas, Louisiana, Mississippi, Alabama and Florida. States featured from the north are identified by the blue curve in Fig. 4a and by blue crosses in Fig. 4c represent the centers of Montana, North Dakota, Minnesota, Vermont, New Hampshire and Maine. Figure 4a presents timelines of ILIs for those two data bands, which indicate that ILIs persist throughout the whole year in both southern and northern bands, but the northern band expresses significantly lower ILI rates. The peaks and trends of both south and north ILI time series are also consistent with that for Albuquerque and New Mexico in general as demonstrated in Fig. S6b.

The emergence of ILIs in Fig. 4a was calculated according to the records of the southern and northern US bands by first approaching a baseline of 1% for the south and 2% for the north. Those are indicated in Fig. 4a by the dotted horizontal blue and red lines respectively. The CDC resource^32^ assigns their own baseline equal to approximately 2.5%. We choose one baseline for the north and one for the south, in consideration of the fact that the ILI rates for the south are approximately twice that for the north. The dates when the ILI percentage rose above those baselines were averaged for the north and south to develop the endpoints for the dotted line in Fig. 4b. This demonstrates that ILI routinely emerges in the southern states about two months earlier than in the northern band. Similarly, the flu season also seems to end earlier in the southern band.

We compared the emergence trend of the ILI pattern for the two state bands to a study by Zhang et al.^36^ of the emergence trend of selected pollens (grass, oak and birch) across the same region. Just as for ILI, the pollens in the southern band of the US emerge 2 months earlier than the pollens in the northern band. Accordingly, the emergence slopes of ILI and of pollen are nearly identical, at approximately 5 days/degree latitude, for both ILI and pollens (grass and oak pollens). Because of the constant slope, the pollen emergence precedes the subsequent SRV emergence by 6 months, regardless of the temperate latitude. If we consider pollens as an antigen (see “Discussions” section), this time lag is consistent with the active lifetime of known immunogens including typical SRV vaccines^37^.

The ILI migration pattern summarized in Fig. 4 challenges earlier explanations of seasonality. One longstanding hypothesis for SRV patterns is a possible connection to sunlight and its related enhancement of vitamin D in humans^2^. Sunlight may be particularly damaging to airborne bacteria through desiccation, and the potential for photolysis to damage both airborne viruses and microbes has been considered in recent literature^38^. However, as shown in Fig. 4, warmer regions emerge into a flu season before colder zones. In addition, it is evident that in the Western US, sunshine remains abundant throughout the winter even as flu outbreaks appear to parallel those in the eastern US. All these observations appear to exclude the ambient temperature or sunlight as a primary controlling factor for the seasonality of respiratory viruses.

## Discussions

### Geostrophic aerosol circulations and SRVS

Although the emergence times and the magnitudes of flus vary from south to north, the peaks of ILIs for both bands also appear to be highly synchronized. The synchrony of peak ILI across some geographic regions, has also previously been noted by Russell et al.^39^, but not directly explained. Those researchers suggested that new virus clades emerge in some locales every year, from which conventional agent-based transmission factors circulate the new strains to various extents around the planet. Given the large spatial separation between the two bands of Fig. 4a, this synchronized oscillation points to a rapid large-scale virus communication between the two bands.

We postulate that this communication is augmented through geostrophic circulation. Seasonal zonal wind diminishment could lead to the sedimentation of SRVs and thus “seed” receptor regions with fresh virus masses. The terminal settling velocity V_*S*_ of a particle in a fluid can be estimated from Stokes law:

1 $$V_{S} = \frac{1}{18}d^{2} g\left( {\frac{{\rho_{p} - \rho_{f} }}{\eta }} \right)$$

where *d* is the diameter of the particle; *g* is the acceleration due to gravity; $\eta$ is the dynamic viscosity; and $\rho_{p}$ and $\rho_{f}$ are the densities of the particle and the fluid (air) respectively. As shown in Table 1, because of the particle size difference, viruses would settle far more slowly than pollen particles in the air, leading to a long residence time for viruses in the atmosphere. Conversely, a much smaller uplifting wind velocity is required to lift virus particles in the air than pollens. The proposed geostrophic circulation mechanism for virus renewal is consistent with our correlation analysis (see Fig. 3b), which identifies the geostrophic wind as an important covariate. After virus seeding from the atmosphere, a conventional SEIR model can then be used to explain the remaining part of virus transmission within a population over that season. The geostrophic circulation and pollen-based notions introduced in this study therefore offer a somewhat challenging but possibly complementary concept to an agent-based model.

The atmospheric seeding concept seems supported by the geostrophic wind pattern observed in the US. The July–August summer monsoonal pattern of Fig. 4c is distinctive, because lower geostrophic winds are coincident with the southern US state band. Given that July follows the season of peak pollination, the lower zonal winds are therefore understood to signify a period of extensive airborne pollen washout and perhaps extensive airborne virus deposition, leading to an earlier Fall viral outbreak in the south. Subsequently, as the Fall geostrophic winds intensify (Figs. S4 and S5), an effective large-scale virus circulation may be re-established, contributing to a large-scale virus circulation and therefore a highly synchronized peak ILI between the two geographic bands as shown in Fig. 4a (see more “Discussions” section below). Therefore, the patterns of SRV outbreak and spread can be consistently explained by considering large-scale virus transport and local-scale pollen cycles.

This large-scale virus circulation may explain observations by Reche et al.^21^ that virus loads monitored in southern Spain often arrive from over the Atlantic Ocean. As noted in the southern band of US states (Figs. 4c and S2–S5), low geostrophic wind levels prevail over the later summer, and those winds are part of a larger atmospheric gyre rooted in the Atlantic Ocean as well. The potential “pooling” of the general atmospheric virus load can be further distinguished in comparison to expected conditions of the northern state locations in Fig. 4c. That northern band is aligned with the well-known North American Jet Stream, evident by the lighter color tones of the map. With stronger lateral winds comes greater lift. Whatever the transport mechanism, and depending on further observations and confirmation, a greater concentration of viral particles across the broad and diverse surfaces of the southern US every summer might then ultimately explain the greater intensity of ILI each winter in the southern band.

This consideration of a viral load fluctuation through changing winds also remains consistent with a pollen causal model, so long as relative residence times and scales are considered. In that scenario, a seasonal load of fresh virus material (perhaps including novel strains) associated with natural annual circulation patterns, may provide a baseline for a subsequent SRV activity, and a subsequent pollen spike can then be attributed to roll that back. Again, this virus-wind explanation is consistent with the study by Reche et al.^21^. Those researchers indicated that viruses appeared to have longer residence times than the larger bacteria. Moreover, they noted a significant sedimentation of virus across their study area in Southern Spain over August. As noted, our Fig. S4 identifies that their geostrophic wind patterns are seasonally low at that time, much like the southern US.

### Antigenic properties of pollen

**Figure 5:**
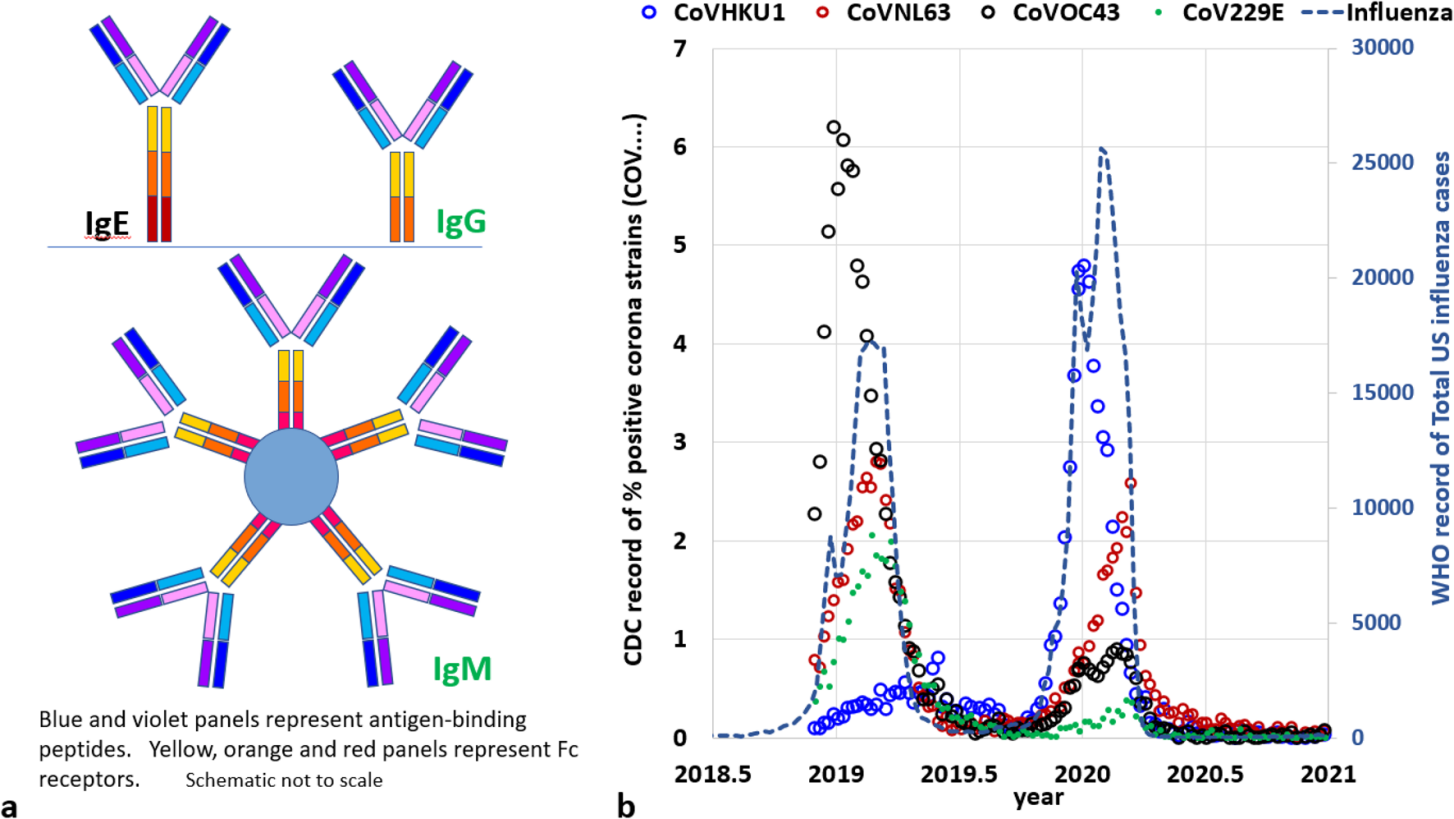
(**a**) Antibody epitope compositions and geometries (not to scale) (**b**) Historical records for the US of 4 separate coronavirus strains (Source: CDC^42^) and all influenza strains (Source WHO^43^).

We noted in the Introduction that although larger than viruses, pollens trigger IgEs and, once the IgE titers in a population rise, this antibody type might also target SRVs. In other words, a virus might not trigger the production of an IgE antibody, but once those antibodies have emerged, they may challenge SRVs. The concept that an IgE antibody could challenge an SRV appears to be largely unexplored, given that the majority of SRV vaccination research and development is tailored towards IgA, IgG, and IgM antibodies. Yet in Fig. 5a which reproduces common representations of antibodies, those epitopes are similar in geometry and composition to the IgE epitope. Moreover, the antigen-binding peptides (blue and violet components), are the same for all, at this level of detail. Table 1 of Roux^40^ and diagrams and immunoelectron micrographs of Roux et al.^41^ further confirm the geometric similarities between IgG, IgA, IgM, and IgE, including dimensions and angular degrees of freedom and flexibilities. Moreover, Sutton et al.^8^ indicates that IgE antibodies may be the most flexible of all types. Finally as noted in the previous sections, the numbers of cases of ILI in temperate latitudes uniformly recede when pollens become airborne. The recession continues in alignment with the half-life of many antibodies, typically covering only a few months^37^. Antigen-triggered IgE antibodies provide a mechanistic explanation for this seasonal phenomenon.

### Environmental, susceptible, infectious and recovered (ESIR) model-

The existing agent-based models and related studies^39^ do not consistently identify any geographical sources of viral loads. Origins of SRV outbreaks are sometimes characterized as zoonotic and attributed to migratory animals such as birds and bats^44^. Those flying creatures happen to have the greatest mobilities and most extensive migrations through the atmosphere, where primary viral loads likely always reside. It may also be remarkable that, for several orders of wild migratory birds, including Anseriformes (including ducks) in North America, and Charadriiformes (including gulls), the prevalence of SRVs has been observed to be highest and lowest at approximately the same months as for humans in North America. In other words, avian flus appear to emerge in early fall and to decline in the following Spring^45^. Thus, avian flu patterns appear to be another manifestation of the proposed geostrophic circulation-pollen cycle regulated viral outbreak and spread mechanism.

By considering large-scale virus transport and pollen-induced immunity, the conventional SEIR model can be modified into a new environmentally influenced transmission model:

2 $$\frac{{\partial C_{v} }}{\partial t} = \mathop{\nabla }\limits^{\rightharpoonup} \cdot \left( {D\mathop{\nabla }\limits^{\rightharpoonup} C_{v} } \right) - \mathop{\nabla }\limits^{\rightharpoonup} \cdot \left( {C_{v} \mathop{V}\limits^{\rightharpoonup} } \right) + V_{s} \frac{{\partial C_{v} }}{\partial z} - \lambda C_{v}$$

3 $$\frac{{\partial N_{S} }}{dt} = - \alpha N_{I} \cdot N_{S} - \beta C_{p} N_{S}$$

4 $$\frac{{\partial N_{I} }}{dt} = \alpha N_{I} \cdot N_{S} + \gamma N_{S} C_{v} |_{z = 0} - \theta N_{I}$$

5 $$\frac{{\partial N_{Im} }}{dt} = \beta C_{p} N_{S} + \theta N_{I} - \mu N_{Im}$$

6 $$N = N_{S} + N_{I} + N_{Im}$$

where *x*, *y* and *z* are the spatial coordinates with *z* pointing upward from the land surface; *C*_*v*_ is the virus concentration in the air; *D* is the dispersion coefficient tensor of a SRV in the geostrosphic layer; *C*_*p*_ is the pollen concentration, determined by local monitoring; $\mathop{V}\limits^{\rightharpoonup}$ is the velocity field of geostrosphic wind; *N* is the total population at geographic location (*x*, *y*); $N_{S}$, $N_{I}$, and $N_{Im}$ are the susceptible, infected and immunized populations, respectively; $\lambda$, $\alpha$,$\beta$, $\gamma$, $\theta$, and $\mu$ are the rate constants for virus decay, human transmission, environmental immunization, infection by geostrophic aerosol virus, human recovery from virial infection, and antibody decay. Equation (1) is subjected to the boundary condition at the land–atmosphere interface (*z* = *0*):

7 $$- D_{z} \frac{{\partial C_{v} }}{\partial z} - V_{s} C_{v} = \omega N_{I}$$

where ω characterizes the release rate of virus to air by the infected population. In this ESIR model, all variables except the virus concentration are treated as local variables. The term, $\gamma N_{s} C_{v} |_{z = 0}$, in Eq. (4) characterizes the infection caused by atmospheric seeding. Given a generally low virus concentration in the atmosphere, this infection is expected to occur only sporadically in both time and space. Equations (2) through (7) can be solved on a global or a regional scale. A full solution of the equations is beyond the scope of this paper. Considering the time-scale disparity between viral recovery (in days) and seasonal viral outbreak cycle (1 year), the time derivatives on the left-hand sides of Eqs. (3) through (5) would vanish. From Eq. (4), we have $N_{I} \propto c_{v}$, that is, the infected population at a location at a given time is proportional to the virus concentration in the atmosphere. For two geographically separated regions, as discussed above, their virus concentrations can rapidly communicate through geostrophic circulation, and as a result the oscillations in the infected population in the two regions should highly synchronize, as observed in Fig. 4a.

### Seasonality of ili and CLI

The applicability of seasonality and pollens to Covid-19 like illnesses (CLIs), which are associated with the SARS-CoV-2 coronavirus strain is of further interest. Price et al.^46^ developed seasonal comparisons for a wide variety of respiratory viruses but did not include coronaviruses due to the lack of a routine testing panel at that time. In any case, the parallel seasonality of CLIs to ILIs has been identified in recent studies^47,48^. Figure 5b further supports that conclusion through an examination of several time series compiled by the CDC for coronaviruses other than SARS-CoV-2, and by the WHO for influenza. The figure identifies a seasonal pattern which appears to be as strong as any such pattern featured in this paper for ILIs. It also demonstrates that for the US, the WHO-reported ILI pattern is relatively synchronous with the CDC coronavirus ensemble.

Additional confidence in the seasonal chart of coronaviruses CoVHKU1, CoVNL63, CoVOC43, and CoV229E is found, not only because coronaviruses are similar in size to influenza viruses (Table 1) but also because all of the series featured in Fig. 5b are based on antigenic tests. Due to their uniquely accurate specificity (as opposed to sensitivity) antigenic tests are typically favored for identifying whether an individual is both ill and contagious with the specific strain^49,50^. Unfortunately, the related practices of sentinel surveillance of almost all respiratory viruses, as exemplified by the Right Size Roadmap (RSR)^51^, were curtailed over recent periods^52,53^. For example, the CDC reported that “Due to the impact of COVID-19 on ILI surveillance, and the fact that the state and territorial epidemiologists report relies heavily on ILI activity, reporting for this system will be suspended for the 2020–21 influenza season”^52^.

We also recognize along with the surveillance literature that the seasonal patterns of many if not all RSVs can sometimes be disrupted by pandemics which emerge at periods outside of the normal cycle. For example, in the case of the 2009 A/H1N1 pandemic, seasonality was initially disrupted. Then in under 2 years, the strain no longer prevailed over the summer in the US^54^. In any case, the potential of a novel strain of SRV to trigger a pandemic is likely to always challenge any surveillance effort. The recent limitations which have been applied to surveillance and to SRV geospatial reporting, may now challenge the completeness and/or accuracy of any near term geospatial updates.

It nonetheless seems appropriate to anticipate that the SARS-CoV-2 strains will ultimately follow the expected seasonal pattern over time, given the other seasonal coronavirus patterns of Fig. 5b. Perhaps this is already the case, given a January 2021 CLI report from the US Department of Health and Human Services^55^. That report of the state-by-state percentages of adult intensive care unit (ICU) beds was also mapped in a recent Wall Street Journal article^56^ to show a clear band of higher CLI cases across the Southern US, spreading from California to Georgia, similar to the ILI pattern shown in Fig. 4a.

In this study, a new hypothesis that relates SRVs to geostrophic circulation coupled with pollen cycles is proposed to address the current gaps in our understanding of ILI and CLI climatological patterns. Given the added but largely unexamined potential that pollens might act as a type of broad-spectrum immunogen, this research points in general to possible improvements to the existing SRV forecasting, prevention and mitigation. More broadly, the potential role of pollens can be viewed as a specific example of a new general concept of environmental immunity in which the ambient environment may endow a local population with a natural resistance to viral infection. Since both environmental immunity and viruses constantly evolve with time, a better understanding of their interplay would be critical for prediction, mitigation and prevention of outbreak and spread of seasonal respiratory viruses including the current Covid-19 pandemic.

## Supplementary Information

Supplementary Information.
