# Supplementary material for: Pollen antigens and atmospheric circulation driven seasonal respiratory viral outbreak and its implication to the Covid-19 pandemic
Source: Sci Rep. 2021 Aug 20;11:16945. doi: 10.1038/s41598-021-96282-y (PMC8379151; doi:10.1038/s41598-021-96282-y)
Supplement: Supplementary file 1 — Supplementary Information. [file 41598_2021_96282_MOESM1_ESM.docx]

**METHODS**

GEOSPATIAL DATA

Averaging and normalization methods were deployed to compare the diverse multiple time series, which often started from sometimes discontinuous daily or weekly records. In some cases, simple summing operations were conducted to integrate multiple feeds of ILI data. Interpolations were applied to intermittent data gaps for some ILI and pollen data sets as well. Our work focused on annual and monthly results. Monthly sets provide 12 data points per year which allows sufficient resolution to capture seasonal inflections.

GEOSTROPHIC DATA

In the geostrophic approximation, each parcel of air starts as a rectangular columnar footprint above the land or ocean surface and extends past the PBL, the tropopause and the stratopause to the top of the atmosphere (TOA), roughly 100 km in altitude. According to the University of California at Riverside (UCAR) resource^M1^ the zonal and meridional geostrophic winds represented in the plates are “the vertical integral of a monthly field as a mass-weighted sum.” The geostrophic units of kg m^-1^s^-1^ directly convert to m s^-1^ From that conversion it is apparent for the plates below (S1 through S5) that the red zones map geostrophic winds of approximately 5.4 m s^-1^, cyan zones depict regions where winds are approximately 2 m s^-1^, and dark blue zones identify the lowest magnitude winds of approximately 0.2 m s^-1^. The streamlines which traverse each plate are included to complement information from the color fields. Black lines originate from 0 deg longitude. Red lines originate from 179.5 deg longitude. Blue lines originate at both + and – 80 deg Lat.

These geostrophic approximations can closely resemble observations of many surface weather features regardless of non-geostrophic boundary layers, shears, vortexes or baroclinicity, even though their fidelity is best under barotropic conditions. Geostrophic simplifications also can sometimes facilitate more direct comparisons of the atmosphere to external variables that otherwise would not be easy or even possible. For example, in Wallace^M2^, the dynamics of global and regional climate systems were geostrophically - simplified to study new correlations of solar cycles to the atmosphere. Not only were high correlations observed between solar cycles and geostrophic parameterizations of wind, moisture and energy, but those relations were also used to develop high accuracy long term forecasts of rivers in selected locations.

Geostrophic datasets are accordingly an important component exercised in this study, not only because their signatures are more likely to capture higher elevation virus transport conditions as well as surface patterns, but also because the UCAR ERAI dataset that is that source, allows for the direct interrogation of any part of the planet’s atmospheric footprint for any month over a recent 36 year period (1979-2014). Surface weather records in comparison are not typically representative of upper altitude weather and are more challenging to assemble outside of major metropolitan regions.

Finally, each geostrophic plate is annotated to identify locations that are featured in this study. Although not explicitly described herein, the city of Wuhan, China is also identified because of its popular association with the SARS-CoV-2 strain. This paper however does not explore any connections between that location and strain.

**SUPPLEMENTAL FIGURES**

The following figures are provided to complement Figure 4c and related aspects of the main paper.


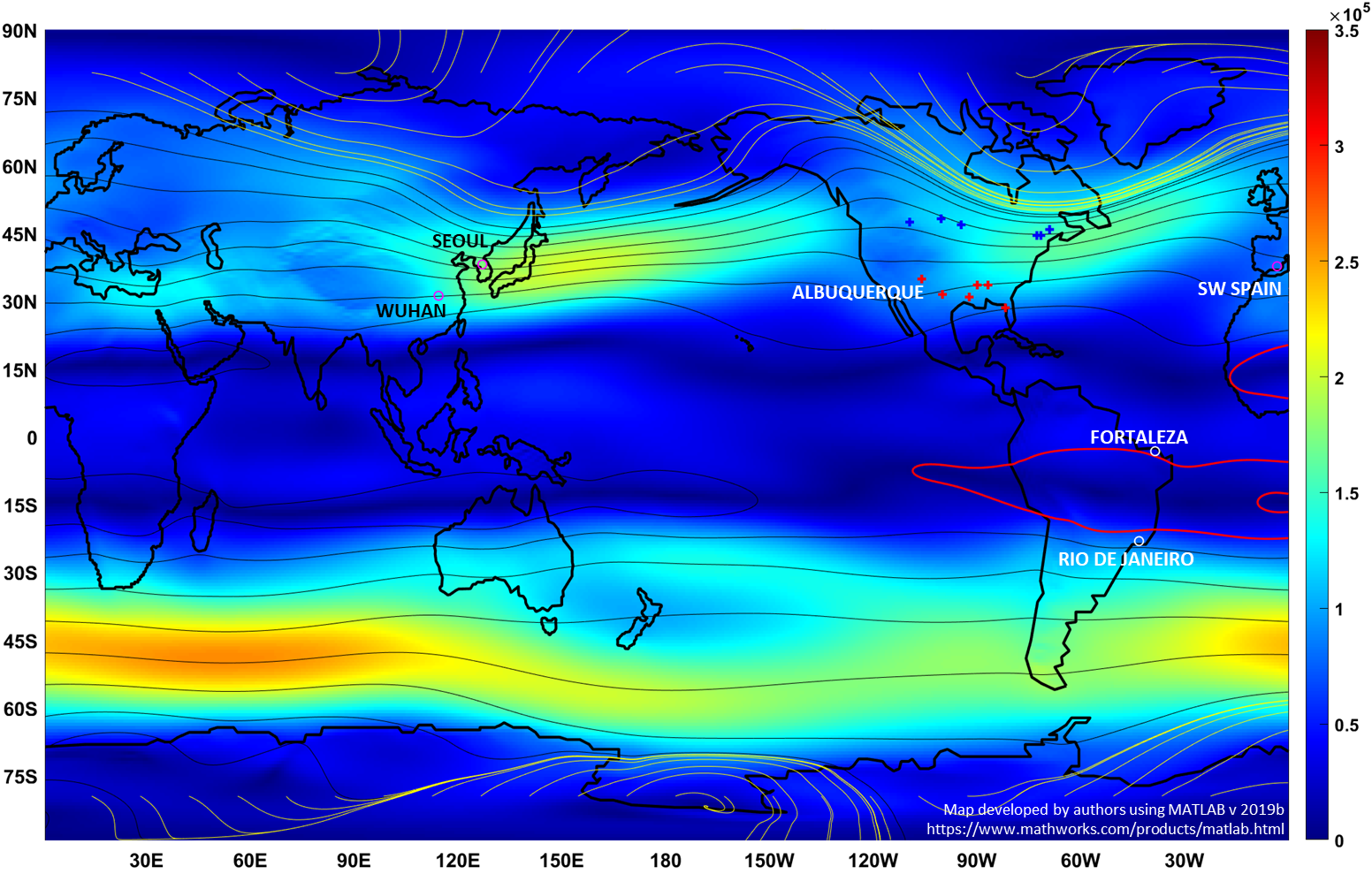


Figure S1. Global Geostrophic Winds Units: kg m^-1^ s^-1^, average of 36 years of UCAR-integrated ERAI monthly records.^M1^ Streamlines: Black lines emerge from west boundary. Red lines emerge from east boundary. Yellow lines originate from 80N and 80S.


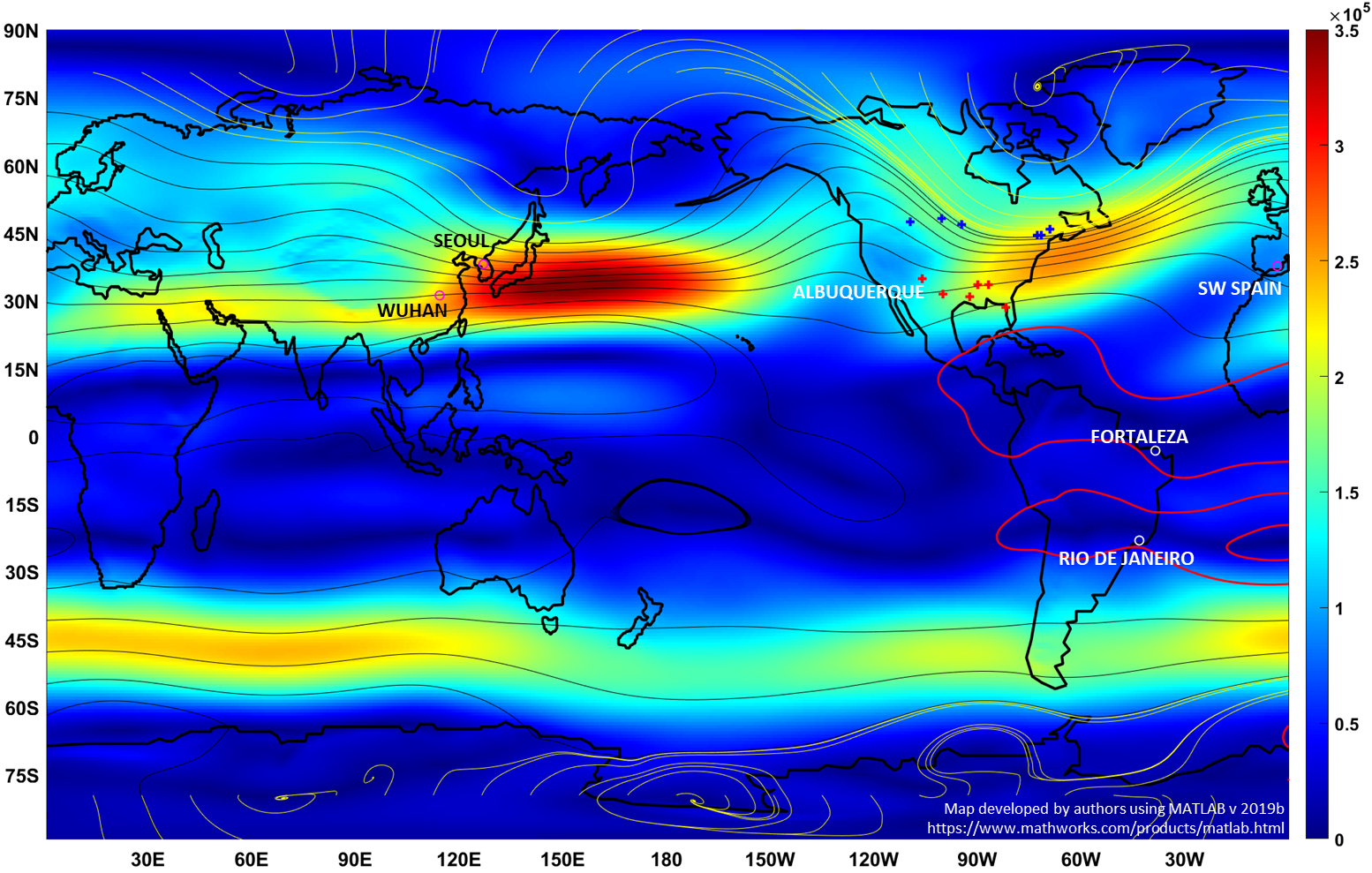


Figure S2. Global Geostrophic Winds kg m^-1^ s^-1^, January Avg from UCAR-ERAI 1979 through 2014.^M1^ Streamlines: Black lines emerge from west boundary. Red lines emerge from east boundary. Yellow lines originate from 80N and 80S


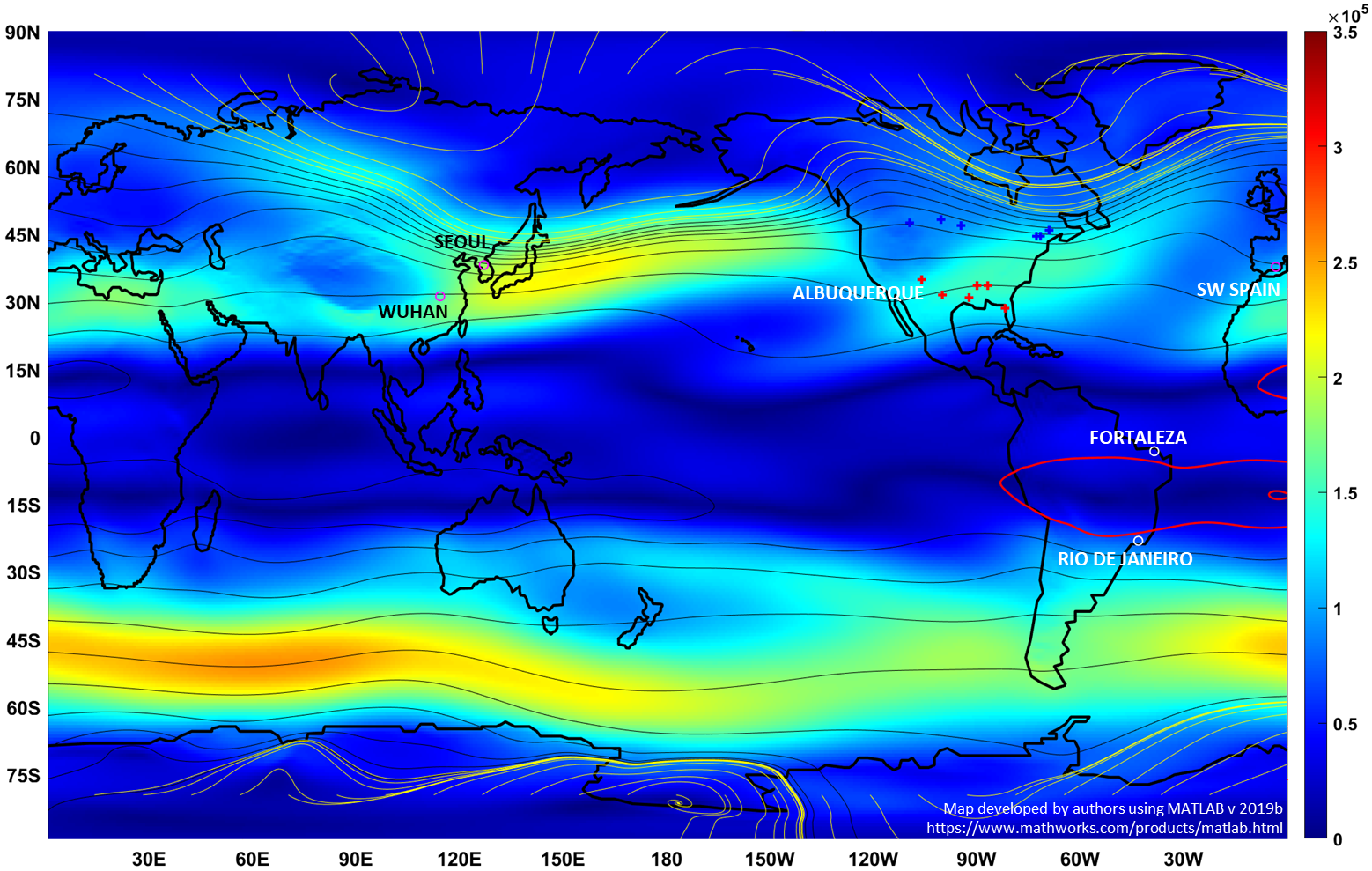


Figure S3. Global Geostrophic Winds kg m^-1^ s^-1^, April Avg from UCAR-ERAI 1979 through 2014.^M1^ Streamlines: Black lines emerge from west boundary. Red lines emerge from east boundary. Yellow lines originate from 80N and 80S.


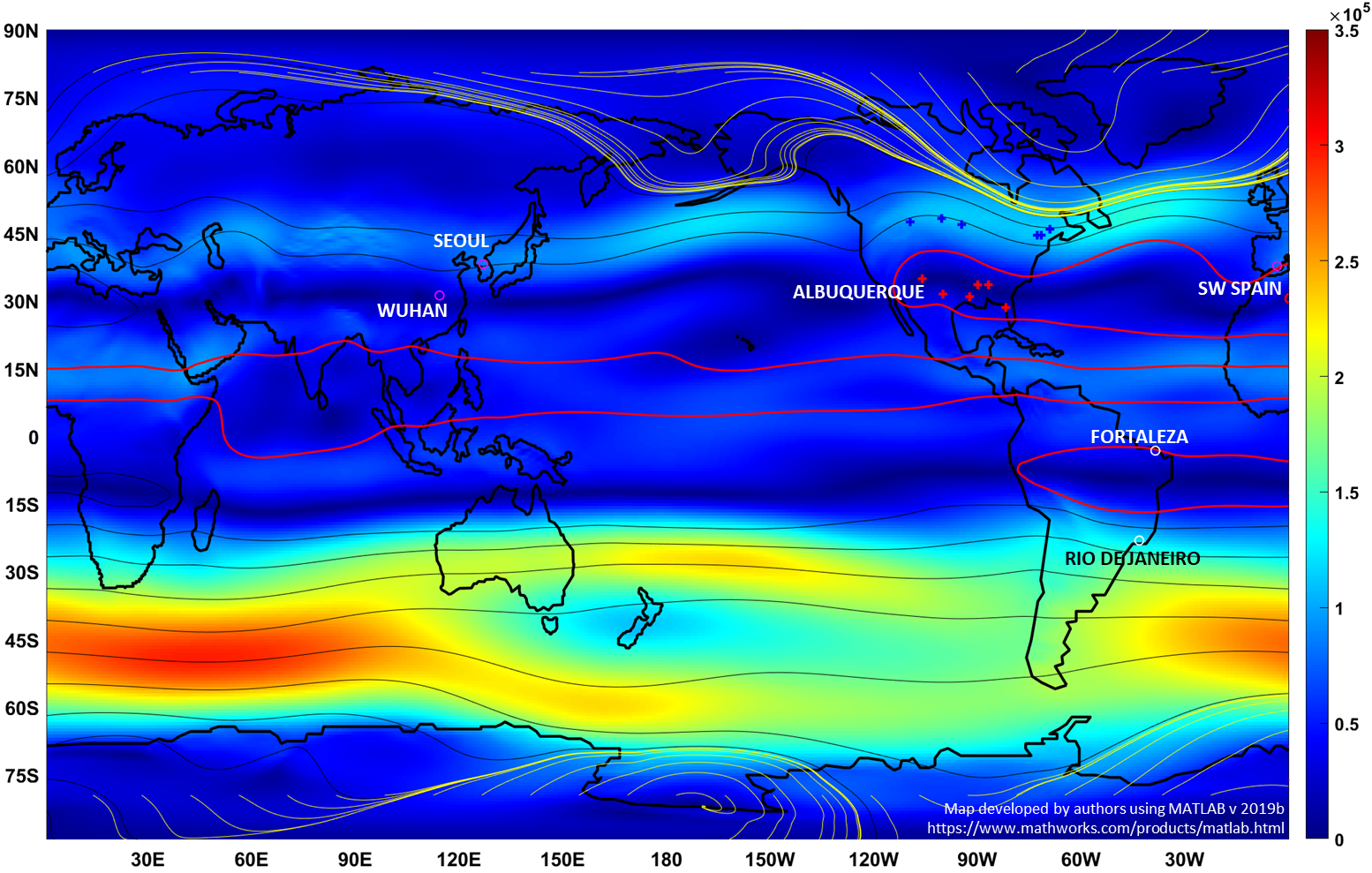


Figure S4. Global Geostrophic Winds kg m^-1^ s^-1^, July Avg from UCAR-ERAI 1979 through 2014.^M1^ Streamlines: Black lines emerge from west boundary. Red lines emerge from east boundary. Yellow lines originate from 80N and 80S.


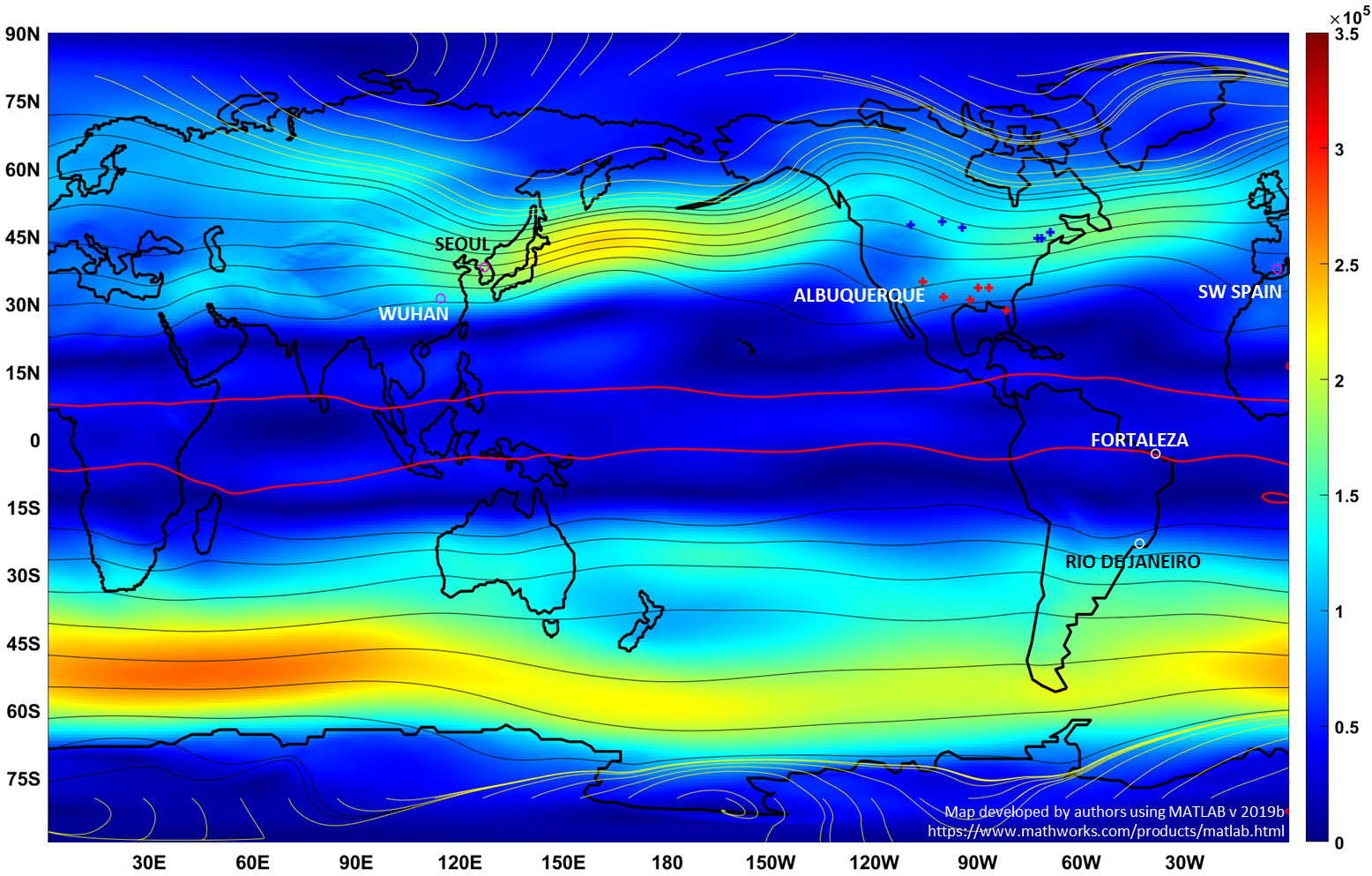


Figure S5. Global Geostrophic Winds kg m^-1^ s^-1^, October Avg from UCAR-ERAI 1979 through 2014.^M1^ Streamlines: Black lines emerge from west boundary. Red lines emerge from east boundary. Yellow lines originate from 80N and 80S.


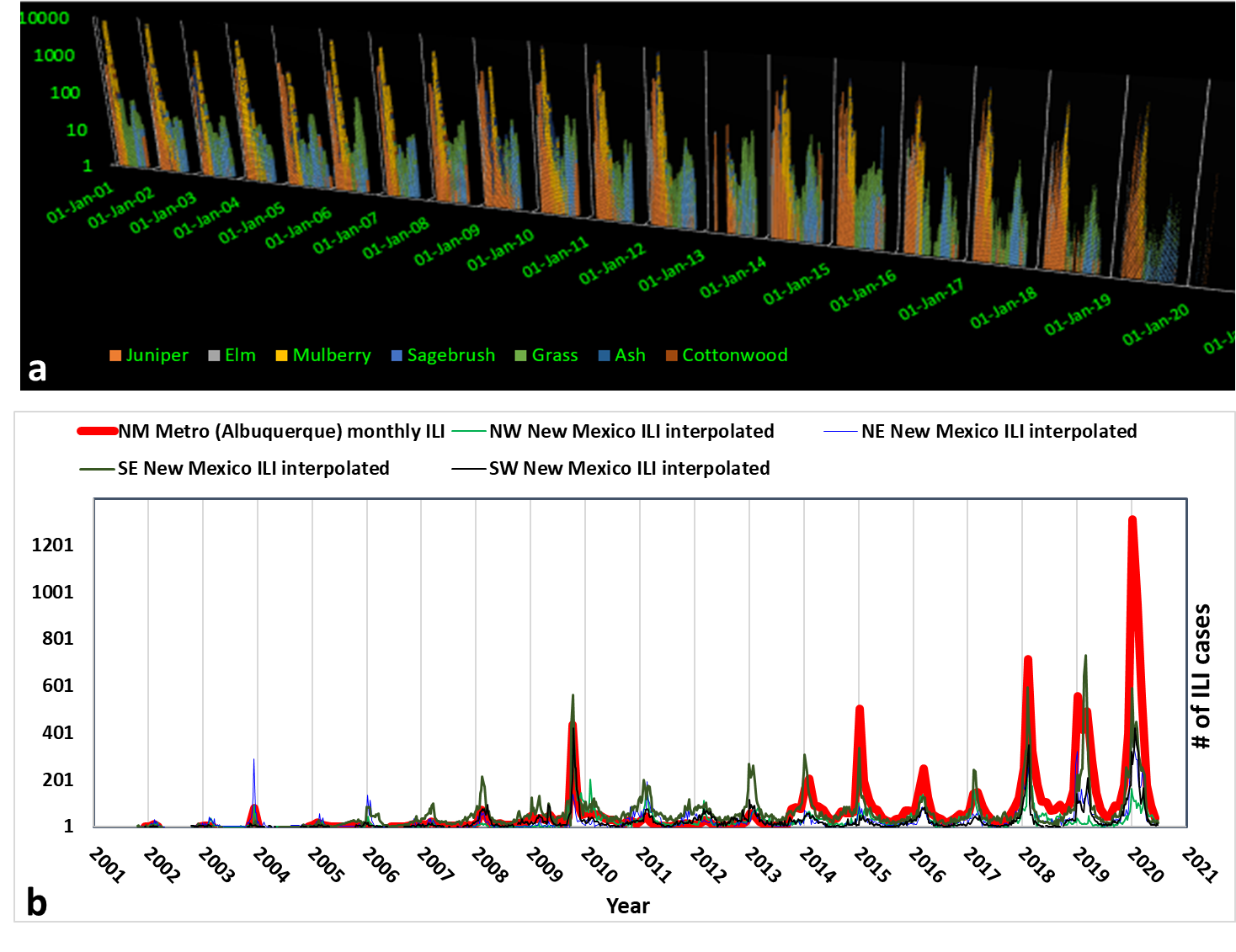


**Figure S6**. a. Selected raw time series from the East pollen monitoring station^M3^ for Albuquerque, NM, US. b. Interpolated ILI values developed from the four NM quadrants and for the Metro (Albuquerque) region^M4^.


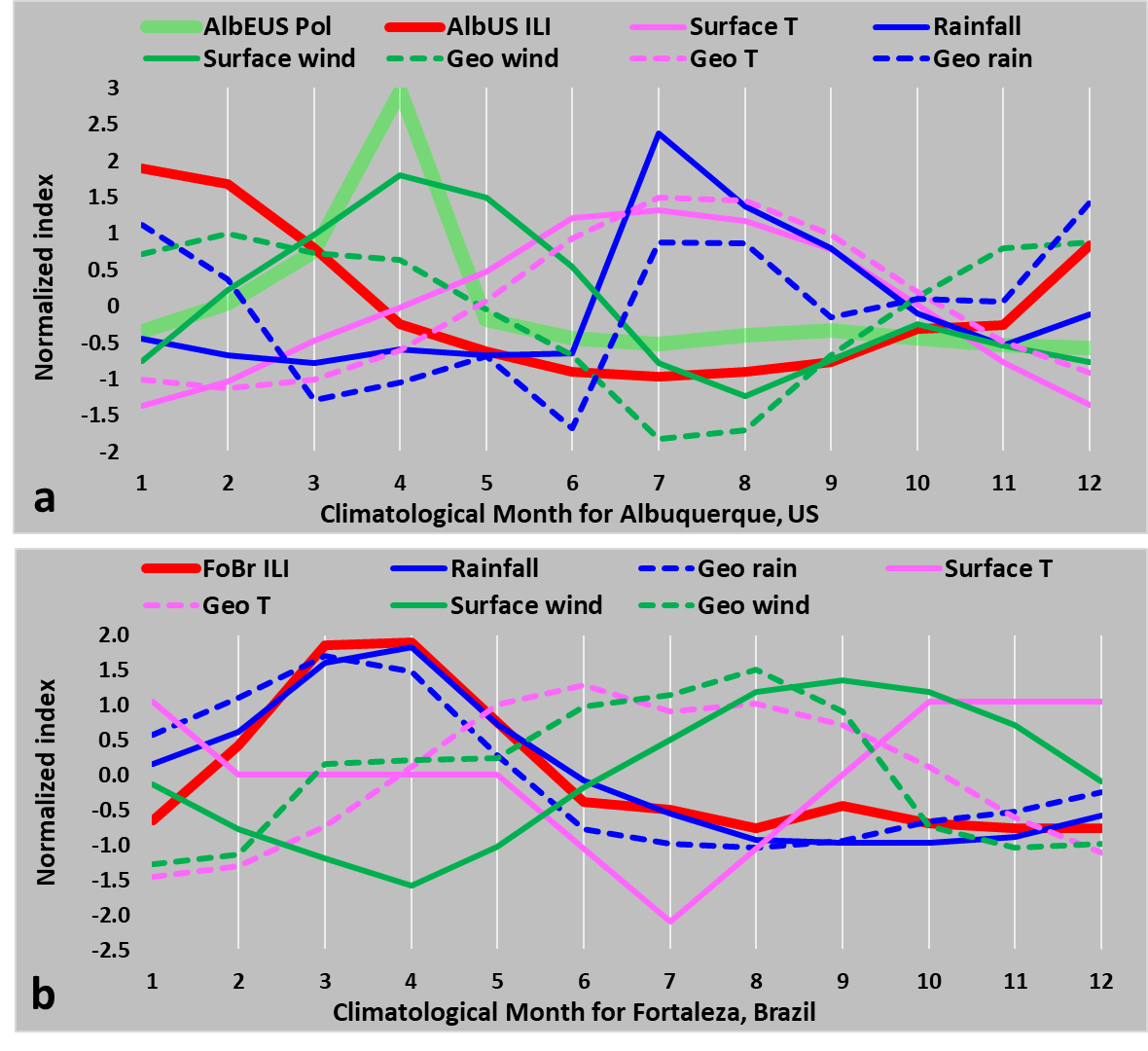


**Figure S7**. Seasonal variations of standardized climatological for two sites in the Western Hemisphere. a. Albuquerque b. Fortaleza

Sources: See geostrophic discussion above and the UCAR resource^M1^ for “Geo” parameters. For surface Albuquerque parameters see online resource^M5^. For surface Fortaleza Parameters see online resources^M6^.

**References for Methods**

M1. University of California at Riverside (UCAR) 2015 integration of ERAI satellite reanalyses products http://www.cgd.ucar.edu/cas/catalog/newbudgets/index.html#ERBEFs – file ‘ERAI.LEDIV.1979–2014.nc

M2. Wallace, M.G., 2019, Application of lagged correlations between solar cycles and hydrosphere components towards sub-decadal forecasts of streamflows in the Western US.   *Hydrological Sciences Journal*, **64**:2m 137-164   doi: 10.1080/02626667.2019.1567925

M3. City of Albuquerque, Environmental Health Department. Air Quality Program. https://www.cabq.gov/airquality (data provided by staff directly).

M4. New Mexico Department of Health. Epidemiology and Response Division. www.nmhealth.org (data provided by staff directly).

M5. Weather.gov site https://www.weather.gov/abq/ (2020)

M6. Commercial weather sites https://weatherspark.com/y/31123/Average-Weather-in-Fortaleza-Brazil-Year- Round and https://weather-and-climate.com/average-monthly(2020)
